# Host Specificity and Spatial Distribution Preference of Three *Pseudomonas* Isolates

**DOI:** 10.3389/fmicb.2018.03263

**Published:** 2019-01-10

**Authors:** Nesli Tovi, Sammy Frenk, Yitzhak Hadar, Dror Minz

**Affiliations:** ^1^Department of Soil, Water, and Environmental Sciences, Agricultural Research Organization–Volcani Center, Rishon LeZion, Israel; ^2^Department of Plant Pathology and Microbiology, Robert H. Smith Faculty of Agriculture, Food and Environment, The Hebrew University of Jerusalem, Rehovot, Israel; ^3^Australian Centre for Ecogenomics, School of Chemistry and Molecular Biosciences, The University of Queensland, St Lucia, QLD, Australia

**Keywords:** isolates, roots, distribution, niche, host, *Pseudomonas*, colonization

## Abstract

Plant hosts recruit and maintain a distinct root-associated microbiota based on host and bacterium traits. However, past studies disregarded microbial strain-host specificity and spatial micro-heterogeneity of the root compartment. Using genetic manipulation, confocal laser scanning microscopy, real-time quantitative PCR, and genome sequencing we characterized the colonization patterns of three *Pseudomonas* spp. isolates native to wheat roots, on the micro-scale. Namely, isolates *P. fluorescens* NT0133, *P. stutzeri* NT124, and *P. stutzeri* NT128. All three isolates preferentially colonized wheat over cucumber roots that served as control for host specificity. Furthermore, not only had the isolates strong host specificity but each isolate had a distinct spatial distribution on the root, all within a few millimeters. Isolate *P. stutzeri*-NT0124 preferentially colonized root tips, whereas *P. fluorescens-*NT0133 showed a preference for zones distant from the tip. In contrast, isolate *P. stutzeri*-NT0128 had no preference for a specific niche on the root. While all isolates maintained genetic potential for motility and biofilm formation their phenotype varied significantly and corresponded to their niche preference. These results demonstrate the importance of spatial colonization patterns, governed by both niche and bacterial characteristics which will have great importance in future attempts to manipulate the plant microbiome by constructing synthetic microbial consortia.

## Introduction

The microbiome of plant roots was shown to have profound effects on growth, nutrition, and health of their plant host. While microbial abundance and diversity in soil are enormous, only specific microbial populations colonize the roots. Root communities are typically less diverse, but of higher abundance than in bulk soil (Uroz et al., 2010; Philippot et al., 2013), mainly due to the contribution of root deposits. Plant type and location on the root affect composition and amount of deposits and the bacterial fraction of the microbiome it hosts (Kravchenko et al., 2003; Jones et al., 2004; Ofek-Lalzar et al., 2014). Recent developments in sequencing technologies expanded our knowledge on root bacterial communities and revealed, to some extent, how plants can influence the composition and activity of their colonizers (Kravchenko et al., 2003; Jones et al., 2004; Ofek-Lalzar et al., 2014; Lareen et al., 2016; Rascovan et al., 2016; Poole, 2017). Despite that, the role of plant host in determining which specific bacteria species colonize its environment is not fully understood.

Colonization was defined as the early step of plant-bacteria interaction (Rodríguez-Navarro et al., 2007; Lugtenberg and Kamilova, 2009). Several important traits offer a selective advantage for bacterial colonization, enabling a bacterium to attach, thrive and compete with others on this unique environment. Among these traits is motility, based mostly on chemotaxis, allowing sensing and reaching the root surface. It has been shown that non-motile or reduced motility mutants are highly impaired in competitive root colonization (Harshey, 2003). Another important bacterial trait enabling an advantage during root colonization is biofilm formation. Most bacteria are found in nature attached to surfaces in multicellular assemblies known as biofilms (Ramey et al., 2004). In the root system, bacteria that are found in biofilm state have several advantages, allowing the organisms that compete in root colonization to create themselves a better protected niche (Lugtenberg et al., 2001).

Past studies have shown that members of the genus *Pseudomonas* are among the most abundant bacteria in root environments of several plant species, particularly those of wheat (Velázquez-Sepúlveda et al., 2012; Ofek-Lalzar et al., 2014; Rascovan et al., 2016). Ofek et al. (2014) showed that root surface microbiomes exhibited a host specific signature effect with specific genetic composition and abundances of bacterial taxa. However, *Pseudomonas* abundance was scarce on cucumber and tomato roots, when grown under identical conditions (Ofek et al., 2014). The specific relationships of *Pseudomonas* species with their host were shown to promote host health and growth (Rainey, 1999; Lugtenberg and Kamilova, 2009; Santoyo et al., 2012). For example, some *P. fluorescens* strains were reported to act as biological control agents against the pathogenic fungus *Gaeumannomyces graminis var. tritic*, responsible for the Take-all disease in wheat (Weller, 1988, 2007; Capper and Higgins, 1993).

The occurrence of several related organisms coexisting in the same niche is a subject for many studies. Little is known about the spatial distribution patterns and preference of roots isolates. The current study seeks to assess colonization specificity of three *Pseudomonas* isolates in two different plant hosts- wheat and cucumber, as well as colonization patterns on the roots. To do so, we isolated several dominant *Pseudomonas* strains from wheat roots and showed that colonization of these Pseudomonads is indeed plant host dependent. We characterized colonization traits such as motility and biofilm formation of the isolates and used confocal laser-scanning microscopy (CLSM) together with real-time quantitative PCR (qPCR) for studying colonization localization and level.

The experiments were designed in accordance to ecological theories, such as “limiting similarity” (MacArthur and Levins, 1967), dictating the number of similar species occupying the same niche and “competitive exclusion principle” when species compete for the same resource (Gause, 1936). We hypothesized that traits such as motility and biofilm formation may indicate which isolate will be a better root colonizer. Lastly, based on previous metagenome studies, we predicted that the *Pseudomona*s isolates would differ in colonization of wheat roots vs. cucumber roots. Our results indeed indicate that these three related *Pseudomonas* isolates differ in spatial distribution on wheat roots: root tips vs. zone distant from the tip, while one isolate did not prefer a specific location.

## Materials and Methods

### Bacterial Stains and Media

The bacterial strains and plasmids that were used in this study are listed in Table 1 and primers used are listed in Table 2. Bacteria were grown on Luria-Bertani broth (LB): 1% Tryptone (Difco Laboratories, USA) 0.5% yeast Extract (Difco Laboratories, USA) and 0.5% sodium chloride (Merck, Germany).These media were also used for biofilm formation, motility, plant colonization, taxonomic classification, and for molecular methods (including: bacterial DNA extraction and plasmid purification). For the purpose of *Pseudomonas* spp. isolation, King's B medium was used [2% peptone (Difco Laboratories, USA), 0.15% heptahydrated magnesium sulfate (Merck, Germany), 0.15%, potassium hydrogen phosphate (Merck, Germany), 0.1% glycerol (BP229-1, Fisher Scientific, USA)]. Solid media was prepared by adding 1.5% Bacto agar to LB or King's B media. Where appropriate, antibiotics were added to maintain or select for plasmids as follows: for *E. coli*, ampicillin (Ap, 171254, Calbiochem, USA) at 100 μg/mL and gentamicin (Gm, G3632, Sigma, USA) at 15 μg/mL and for all *Pseudomonas* isolates, Gm at 30 μg/mL.

**Table 1:** List of strains and plasmids used in this study and their source.

**Strains**	**Relevant genotype or sequence**	**Source or reference**
*P. aeruginosa* PAO1	Wild type	Holloway, 1955
*Pseudomonas stutzeri* NT0124	Isolated from wheat roots- PRJNA273703	This research
*Pseudomonas stutzeri* NT0128	Isolated from wheat roots- PRJNA275697	This research
*Pseudomonas fluorescens* NT0133	Isolated from wheat roots- PRJNA275699	This research
NT0124/pBT270: miniTn7T-Gm-GFP	Ap^r^ and Gm^r^, pUCP18-miniTn7T2.1 -GFP	This research
NT0128/pBT270: miniTn7T-Gm-GFP	Ap^r^ and Gm^r^, pUCP18-miniTn7T2.1 -GFP	This research
NT0133/pBT270: miniTn7T-Gm-GFP	Ap^r^ and Gm^r^, pUCP18-miniTn7T2.1 -GFP	This research
**PLASMIDS**		
pBT270/ *pUCP18-miniTn7T2.1Gm- GFP*	Ap^r^ and Gm^r^, Mini-Tn7-gfp(mut3). Integration vector for gfp.	Zhao et al., 2013
Ptns2	Ap^r^; helper strain for mobilizing miniTn7 into *P. aeruginosa* strains by mating	Choi and Schweizer, 2006
pGEM:*tef*	pGEM::*tef, Ap^*r*^*	This research

**Table 2:** List of primers used in this study and their source.

Plant tef_f	ACTGTGCAGTAGTACTTGGTG	Ruppel et al., 2006
Plant tef_r	AAGCTAGGAGGTATTGACAAG	Ruppel et al., 2006
GFP_RT_ f	CACTGGAGTTGTCCCAATTC	This research
GFP_RT_r	GGCCATGGAACAGGTAGTTT	This research
F311Ps_f	CTGGTCTGAGAGGATGATCAGT	Milling et al., 2005
Ps-rev_r	TTAGCTCCACCTCGCGGC	Widmer et al., 1998
rpoB_4042-4062_R	GATGTTYTTGTACATCTTGG	This research
rpoB_3159-3178_F	GACAAGTTYGARGACAAGAAG	This research

### Isolation, Identification, and Phylogenetic Analysis of Wheat Root *Pseudomonas* spp.

Wheat seeds (*Triticum turgidum* cv. Negev) were surface-sterilized by soaking in 3% sodium hypochlorite for 1.5 min, followed by 70% ethanol for 1.5 min, and three washes with water. The sterilized seeds were planted in sandy loam soil (81% sand, 6% silt, and 13% clay) obtained from Maon region in the Negev, Israel (31.21 N 34.27 E).The seeds were germinated and grown at 25°C for 12 days, until first true leaf appeared. Plants were irrigated with half-strength Hoagland solution when needed (Ofek et al., 2014). After 12 days, the plants were carefully removed and the roots were separated from the shoots and washed in sterile saline 3 times. The roots were then vortexed for 30 min in 10 ml saline (0.85% NaCl) to extract adhering bacteria. The roots were then removed and serial dilutions of the remaining saline was plated on King's B medium for isolation of *Pseudomonas* species (King et al., 1954). Strain identification as belonging to the genus *Pseudomonas* was confirmed by PCR using two sets of *Pseudomonas* specific primers listed in Table 2 (F311Ps_f, Ps-rev_r, rpoB_4042-4062_R, rpoB_3159-3178_F) and by sequencing parts of 16S rRNA and *rpoB* genes using Sanger sequencing (MCLAB, San Francisco, USA). Resulting sequences were compared to the nr database using online BLAST tool. Enterobacterial repetitive intergenic consensus sequences (ERIC) PCR was performed to cluster identical isolates, as described previously (De Bruijn, 1992).

### Taxonomic Classification of Isolates, Gene Calling, and Annotation

Taxonomic classification of isolates was done by sequencing the genome of each isolate. DNA was purified using Exgene extraction kit (GeneAll, Korea) and prepared for sequencing using Nextera DNA sample preparation kit FC-131-1024 (Illumina, USA). *De novo* sequencing was performed on an Illumina MiSeq. Sequence data was quality trimmed using Trimmomatic (Bolger et al., 2014) and assembled using ABySS v. 1.5.2 (Simpson et al., 2009). The genomes are available on NCBI (BioSample: SAMN03295272, SAMN03352191, SAMN03352192). Phylogenetic classification of isolates was determined by concatenation of 120 phylogenetically informative proteins as previously described (Parks et al., 2017). Shortly, the completeness and contamination of the selected genomes was estimated using CheckM (Parks et al., 2015). These genomes were then subjected to gene calling by Prodigal (Hyatt et al., 2010) and search for the 120 phylogenetic informative proteins which were then aligned, both search and aliment were done by HMMER v.3.1b2 (Eddy, 1998). Trees were inferred with FastTree v.2.1.7 under WAG+ GAMMA models and decorated, rooted and bootstrapped using 100 non-parametric bootstrap replicates by GenomeTreeTk (https://github.com/dparks1134/GenomeTreeTk).

### Molecular Methods

Total DNA was extracted from 0.25 g roots using soil GeneAll kit (Geneall, Korea, soil DNA production kit, 114150) according to the manufacturer's protocol. Genomic DNA was extracted using GeneAll kit according to the manufacturer's protocol (Geneall, Korea, Genomic DNA purification kit, K0512), plasmids were purified with the QIAprep Spin Miniprep Kit (QIAGEN, Germany). All primers were obtained from Integrated DNA Technologies (IDT). For quantifying plant gene copy number by qPCR, we used known DNA concentration of a specific plasmid containing the target region coding for translation elongation factor 1 (*tef* ); pGEM:*tef*. For this purpose PCR primers listed in Table 2 for the *tef* region were used to amplify a 150-bp PCR product from wheat DNA as a template. To confirm the specify of amplification, the PCR product was analyzed by gel electrophoresis, and cloned into pGEM vector using pGEM cloning kit according to the manual (pGEM-T Vector, Promega, WI, USA).

Construction of chromosomally GFP-expressing strains the vector *pUCP18-miniTn7T2.1Gm- GFP* (Zhao et al., 2013) was used to chromosomally insert *GFP* to strains: NT0124, NT0128, NT0133. The vector was introduced together with pTNS2 helper plasmid into *Pseudomonas* isolates (NT0124, NT0128, and NT0133) by transformation as described by Choi and Schweizer (2006). Briefly, overnight culture of each strain was grown in LB medium at 30°C with shaking (225 rpm). The next day, culture was divided into four Eppendorf tubes and centrifuged at 16,000 g and room temperature for 2 min. The supernatant was discarded and cell pellet was suspended in 300 mM cold sterile sucrose. This step was repeated 4 times. The electrocompetent cells were transferred to electroporation cuvette and equal amounts (100 ng) of the vector and helper plasmid were added to the cells. After electroporation, the cells were place in LB medium and incubated with shaking at 30°C for 1 h for recovering. After recovering, the cells were plated on LB plates supplemented with Gm antibiotics. The colonies obtained were confirmed using PCR which targeted the GFP gene as well as using fluorescence microscope (Choi and Schweizer, 2006; Zhao et al., 2013; Marmont et al., 2017).

### Motility Experiments

Swimming plates contained 1% tryptone (Difco Laboratories, USA), 0.5% NaCl (Merck, Germany), and 0.3% agar (Difco Laboratories, USA). Bacteria were taken with a toothpick from colonies grown overnight on LB plates and inoculated onto swimming plates by stabbing the agar with the toothpick midway and incubated at 30°C for 24 h. Total of 8 different plates were used to calculate swimming potential by measuring the diameter of the bacterial colony formed. Twitching plates contained 1% tryptone (Difco Laboratories, USA), 0.5% yeast extract, 1% NaCl (Merck, Germany), and 1% agar (Difco Laboratories, USA). Bacteria were taken from colonies grown overnight on LB plates and inoculated onto twitching plates by stabbing the agar with the toothpick all through the agar and inoculated at 30°C for 24 h. Total of 10 replicates were used to calculate twitching potential by measuring diameter of bacterial spread at the bottom of the plate after removing the agar and dying the bacteria at the bottom using crystal violet 1% (c0775, Sigma, USA).

### Static Biofilm Assay

Overnight grown liquid cultures were diluted 1:100 in LB and 100 μL was transferred to each well in polystyrene 96-well plate (Nunclon, 167008, Nunc Brand Products, Denmark) and incubated at 30°C for 20–24 h. To measure biofilm formation in each well, plates were cleared of planktonic bacteria by double washing with water, filled with 150 μL aqueous solution of 1% crystal violet (c0775, Sigma, USA) and incubated at room temperature for 15 min. Unbound crystal violet was washed with water and biofilm-bound was extracted with 200 μL ethanol (AC615090010, Fisher Scientific, USA). Hundred Microliter of the extracted crystal violet was transferred into a new 96-well plate and assessed by measuring the OD at 600 nm using plate-reader (iMark microplate reader, Bio-RAD, USA). Total of 24 wells repeats were analyzed for each isolate and 16 for PAO1.

### Plant Growth and Bacterial Colonization

Wheat seeds (*T. turgidum* cv. Negev, Hazera, Israel) or cucumber seeds (*Cucumis sativus* cv. Kfir, Zeraim Gedera, Israel) were sterilized as described above. The sterilized seeds were cultivated in sterile mix of sandy loam soil with perlite 9:1 (w/w), hydrated with half-strength Hoagland solution (Ofek et al., 2014). For soil inoculation, bacteria were grown in LB overnight, diluted 1:100 in LB and grown for additional 3 h and washed 3 times with saline, then inoculated at 10^6^ bacteria per gram soil-perlite mixture. Plants were grown for 10–12 days. After the growth period, the roots were carefully removed from the pots and washed in sterile saline and soil adhering to the roots was removed by vortex. Excess saline was removed from roots by putting them briefly on sterile filter paper and roots were weighted and used for either DNA extraction and qPCR or visualizing the bacterial colonization by confocal laser scanning microscopy.

### Confocal Laser Scanning Microscopy (CLSM)

Roots were stained with Hoechst 3334 (Nucblue, R37605, Thermo Fisher, USA) according to the manufacture protocol. Images were acquired using either OLYMPUS IX 81 (Olympus Corporation, Japan) inverted laser scanning confocal microscope (FLUOVIEW 500) equipped with a 405 and 488 laser lines and a 20 × 0.7 NA UPlanApo objective, or Leica SP8 laser scanning microscope (Leica, Wetzlar, Germany), equipped with a solid state lasers with 405 and 488 nm light and HC PL APO CS2 20x/0.75 objective (Leica, Germany) and Leica Application Suite X software (LASX, Leica, Germany).

### Real-Time PCR Quantification (qPCR) of Root Colonization

Average of 0.25 g of cucumber or wheat roots was collected from three different plants grown in the same pot for DNA extraction, as described above. Minimum of eight different replicates (individual pots) of each plant were used for quantifying the abundance of colonized isolates in root samples. Bacterial abundance on roots was quantified by targeting GFP (labeled isolate) and normalizing it to the copy number of the plant *tef* gene (pGEM:*tef* contained the *tef* region, coding for translation elongation factor). In all samples, GFP target numbers were divided by the *tef* target number, as described by Ofek et al. (2011). The primers used for quantifying each gene are listed in Table 2. Each gene copy numbers were calculated by StepOne software v2.3 (Applied Biosystems, CA, USA) using known DNA concentration and the specific plasmid (Table 2) plus insert molecular weight, estimated from their lengths. All qPCR assays were conducted in polypropylene 96-well plates and were performed using a StepOne Plus real-time PCR system (Applied Biosystems, USA). Plasmids DNA concentration was measured using Qubit fluorometric quantification (Thermo, Fisher scientific, USA) and Qubit dsDNA BR assay kit (Thermo, Fisher scientific, USA ,Q32853). Eight fold dilution series of the plasmids pGEM:*tef* and pBT270 and *pUCP18-miniTn7T2.1Gm- GFP* were conducted from 10^∧9^ copies per 1 ml to 10^∧2^ copies per 1 ml. The standards and each sample within each treatment were tested in triplicates. The slope of the standard curve, correlation coefficient, and amplification efficacy were calculated using StepOne software v2.3. Reaction conditions were as follows: each 20 μL reaction contained: 10 μL Absolute Fast SYBR Green Master Mix (AB-4385612, Thermo, Fisher scientific, USA), 0.6 μL of each primer (100 μM), 7.8 μl H_2_O, and 1 μL template DNA (diluted 1:10). PCR conditions were: 5 min at 95°C, followed by 40 cycles of 95°C for 5 s, 60°C for 30 s. Melting curve analysis of the PCR products was conducted following each assay to confirm that the fluorescence signal originated from specific PCR products.

### Statistical Analysis

Statistical analysis was done using JMP 13 software (Sall et al., 2012). Normal distribution of data was evaluated using the Shapiro–Wilk test for determining the use of parametric (*p* > 0.05) or non-parametric (*p* < 0.05) tests. Isolates biofilm formation capabilities was evaluated by comparing OD_600_ values using unpaired *t*-test with *p* = 0.05 as threshold for rejecting the null hypothesis. Differences in motility rates, both swimming and twitching, were determined by comparing the diameter of the bacterial colony formed using the non-parametric Wilcoxon test. Finally, values resulting from the qPCR analysis determining colonization of isolates between plant types was analyzed using two way analysis of variance (ANOVA, *p* < 0.005, *F* ratio = 8.6).

## Results

### Isolation of Wheat Root *Pseudomonas* Populations

**Figure 1:**
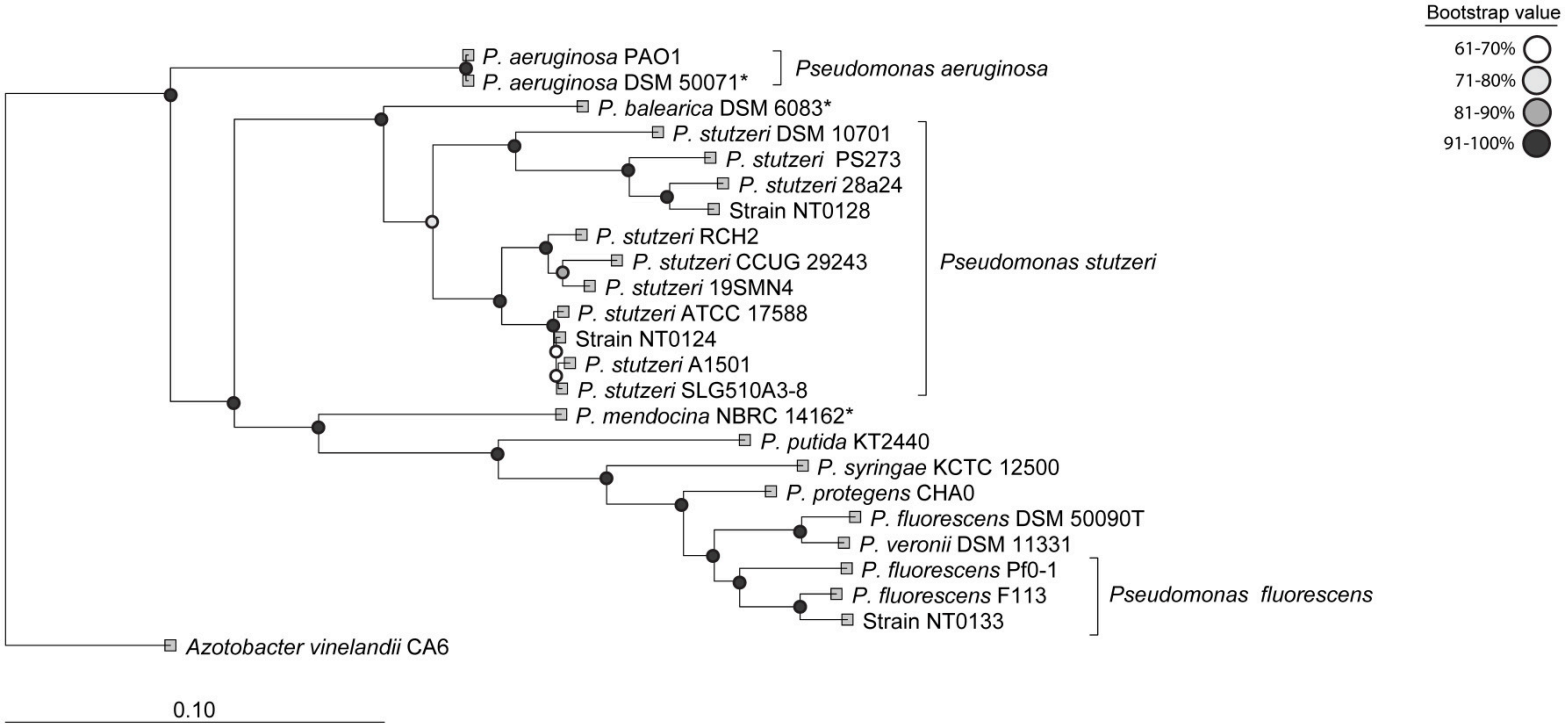
Phylogenetic characterization of the three *Pseudomonas* isolates. The taxonomic annotation was done using 20 well-studied *Pseudomonas* strains as reference. The tree was inferred by the concatenation of 120 phylogenetically informative proteins (Parks et al., 2017). The names used are according to NCBI taxonomy and type strains are indicated by *.

We isolated approximately 70 putative *Pseudomonas* strains from wheat roots, on King's B medium. Their affiliation with the *Pseudomonas* genus was confirmed by PCR using two sets of *Pseudomonas*-specific primers: one targets the *Pseudomonas* 16S rRNA gene (Widmer et al., 1998; Milling et al., 2005) and another that targets *Pseudomonas rpoB* gene (Table 2). Of the 70 isolates, only 30 were positively identified by both *Pseudomonas*-specific primer sets. These isolates were clustered into four groups, based on morphology and Enterobacterial Repetitive Intergenic Consensus (ERIC) PCR patterns (De Bruijn, 1992). Converging evidence, based on ERIC PCR and *rpoB* gene sequencing, suggested that most of the isolates were closely related to *P. stutzeri*. Three isolates, namely NT0124, NT0128, and NT0133, were selected as representatives of the three main wheat *pseudomonas* populations (one group had only one isolate, which did not survive in cultivation). The taxonomic inference of the three isolates was performed by the concatenation of 120 proteins previously found to be phylogenetic informative (Parks et al., 2017). Results showed that each of the isolates was phylogenetically distinct (Figure 1). Two isolates (NT0124, NT0128) were annotated as two strains of *P. stutzeri* and one (NT0133) as a strain of *P. fluorescens*. To gain more information regarding differences between the isolates we characterized several traits known to be important for plant root colonization. Comparing unique and shared orthologs predicted open reading frames (ORFs) showed that each of the isolates share between 53.8 and 65.1% ORF, with only 11.2–34.6% unique ORF (Figure S1).

### Motility and Biofilm Formation

**Figure 2:**
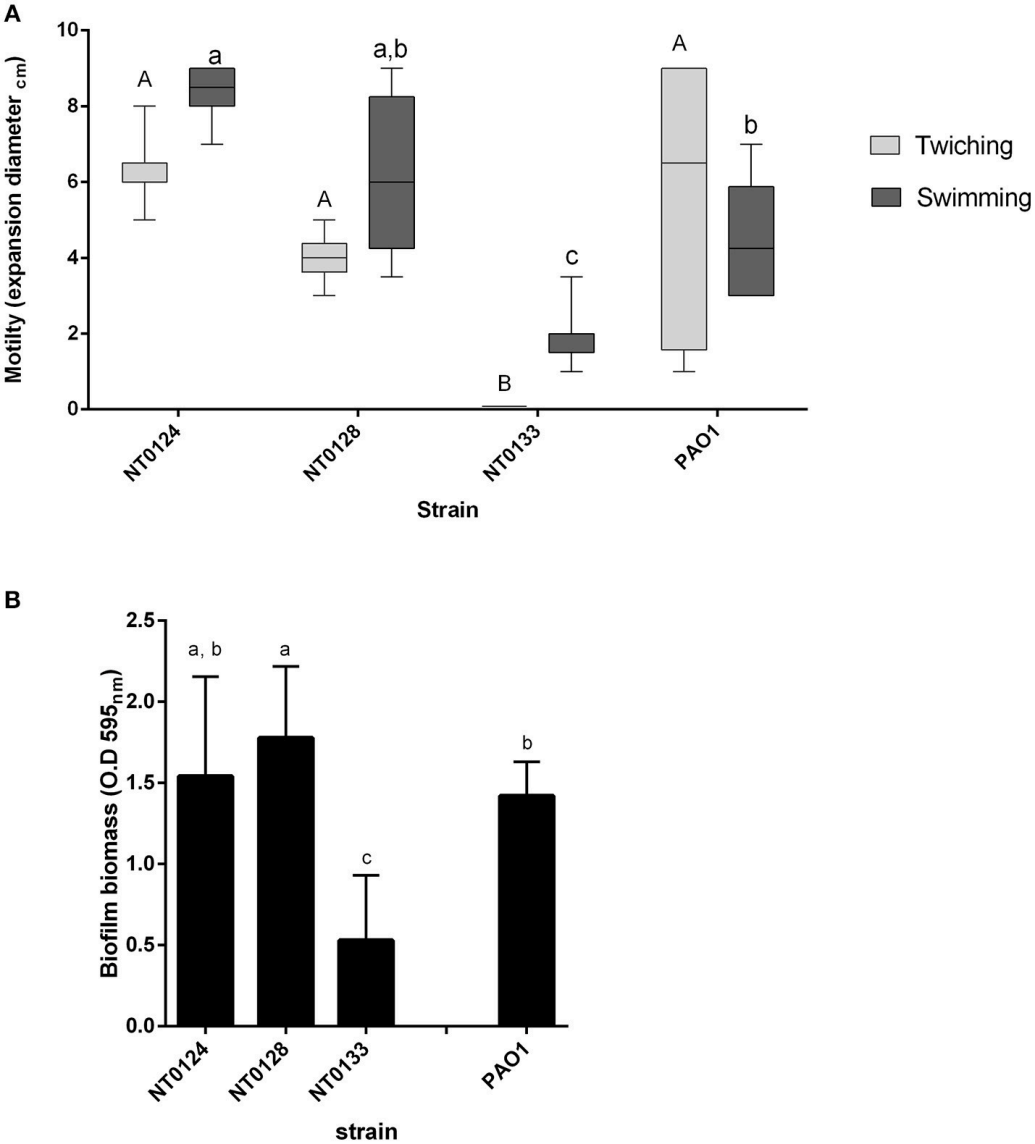
Motility and biofilm formation of isolates NT0124, NT0128, NT0133, and the reference bacteria PAO1. **(A)** Motility levels of the isolates, measured as colony expansion zone diameter formed after 24 h of growth on agar plates [in light gray: twitching motility (*n* = 8) and in dark gray: swimming motility (*n* = 10)]. The expansion zones in each motility type were significantly different between each of the strains levels not connected by the same letters are significantly different A-B and a-c (*p* < 0.0002). Isolate NT0133 did not show twitching ability. Error bars indicate standard deviation and bar line represents average. **(B)** Biofilm biomass formed by the isolates. Error bars indicate standard deviation. Isolates NT0124, NT0128, and PAO1 were significantly different than NT0133 [*p* < 0.0001, (*n* > 16) levels not connected by the same letters are significantly different a-c].

Motility has an important role in biofilm formation and host colonization (O'toole and Kolter, 1998; Mattick, 2002). The three isolates were tested for two types of motility: swimming (involves flagella) and twitching (mediated by type IV pili) and expansion zone was used to measure both types (Mattick, 2002). *P. aeruginosa* PA01 served for comparison and as a positive control for motility and biofilm formation, as this strain is often used as model bacteria for these traits (Costerton et al., 1999; Mattick, 2002; Sriramulu et al., 2005). Both isolates NT0124 and NT0128 surpassed isolate NT0133 motility performances (Figure 2A). Twitching motility of isolates NT0124 and NT0128 was found to be at a similar level to that of PAO1, at a diameter of around 6–8 mm. Isolate NT0133, on the other hand, had impaired twitching motility activities compared to NT0124 and NT0128 (*p* < 0.0001) and did not show twitching ability. Strain NT0124 was found to be the most superior swimmer, 1.5 times faster than PAO1 (*p* < 0.0002). NT0133 was significantly less motile than NT0124 (*p* < 0.0001) and NT0128 (*p* < 0.0002) (Figure 2A).

The three selected isolates were examined for biofilm formation ability by the commonly used crystal violet assay (O'Toole, 2011; Coffey and Anderson, 2014). All three strains formed biofilm to some degree (Figure 2B). However, isolates NT0124 and NT0128, both showed hyper-biofilm phenotype, in contrast to isolate NT0133 which formed three times less biofilm under the same conditions. Isolate NT0124 biofilm level was similar to that of *P. aeruginosa* PA01, and NT0128 biofilm levels were even higher (*p* value for NT0133 and NT0124 *p* < 0.0001, t Ratio = −7.3, for NT0133 and NT0128 *p* < 0.0001, t Ratio = −9.2, for PAO1 and NT0128 *p* < 0.001, t Ratio = −2.4 and for PAO1 and NT0133 *p* < 0.0001, t Ratio = 6.1).

### *Pseudomonas* Isolates Patterns of Root Colonization

**Figure 3:**
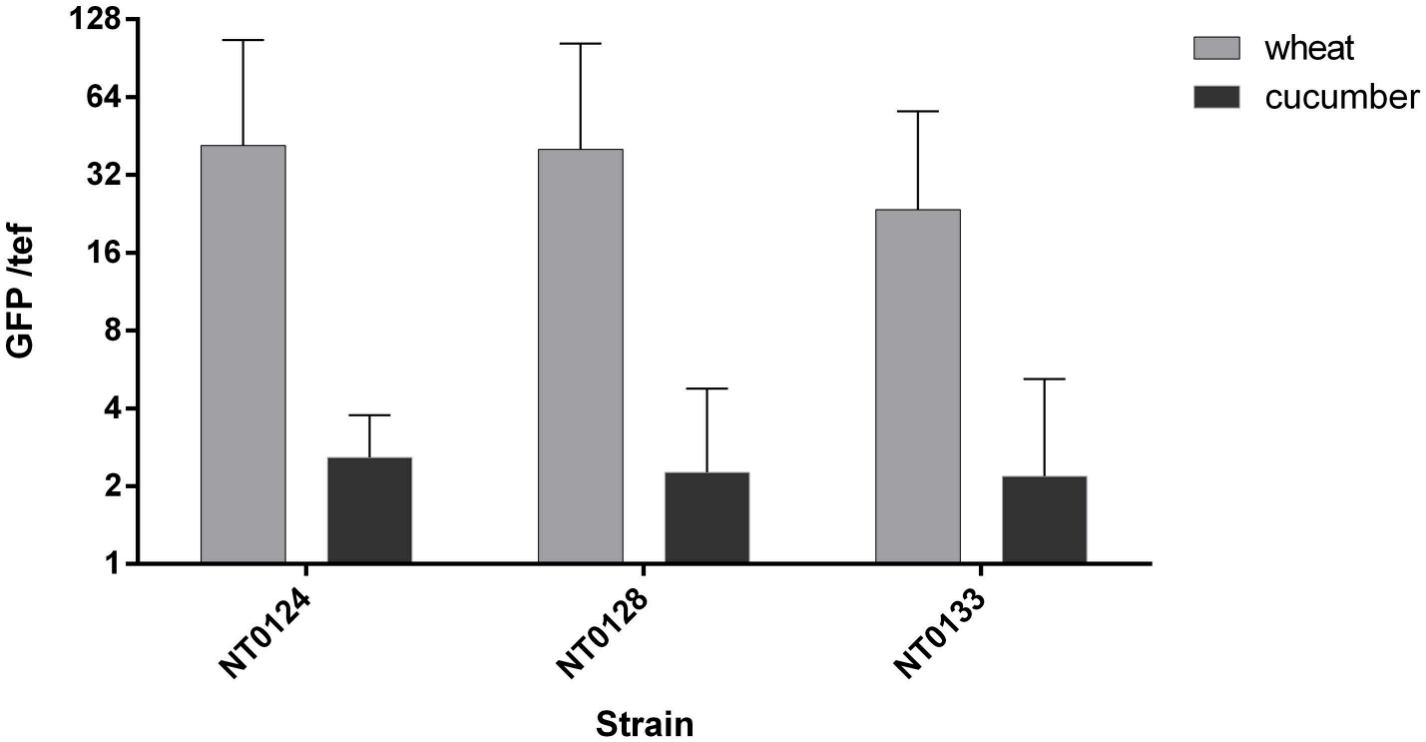
Wheat and cucumber root colonization by the isolates. Real-time qPCR analysis was conducted by quantifying GFP copy numbers and normalizing by plant *tef* copies. Error bars indicate standard deviation (*n* > 8). Two way ANOVA analysis revealed significant difference between abundance of all isolates as affected by the plant type (*p* < 0.005, F ratio = 8.6).

**Figure 4:**
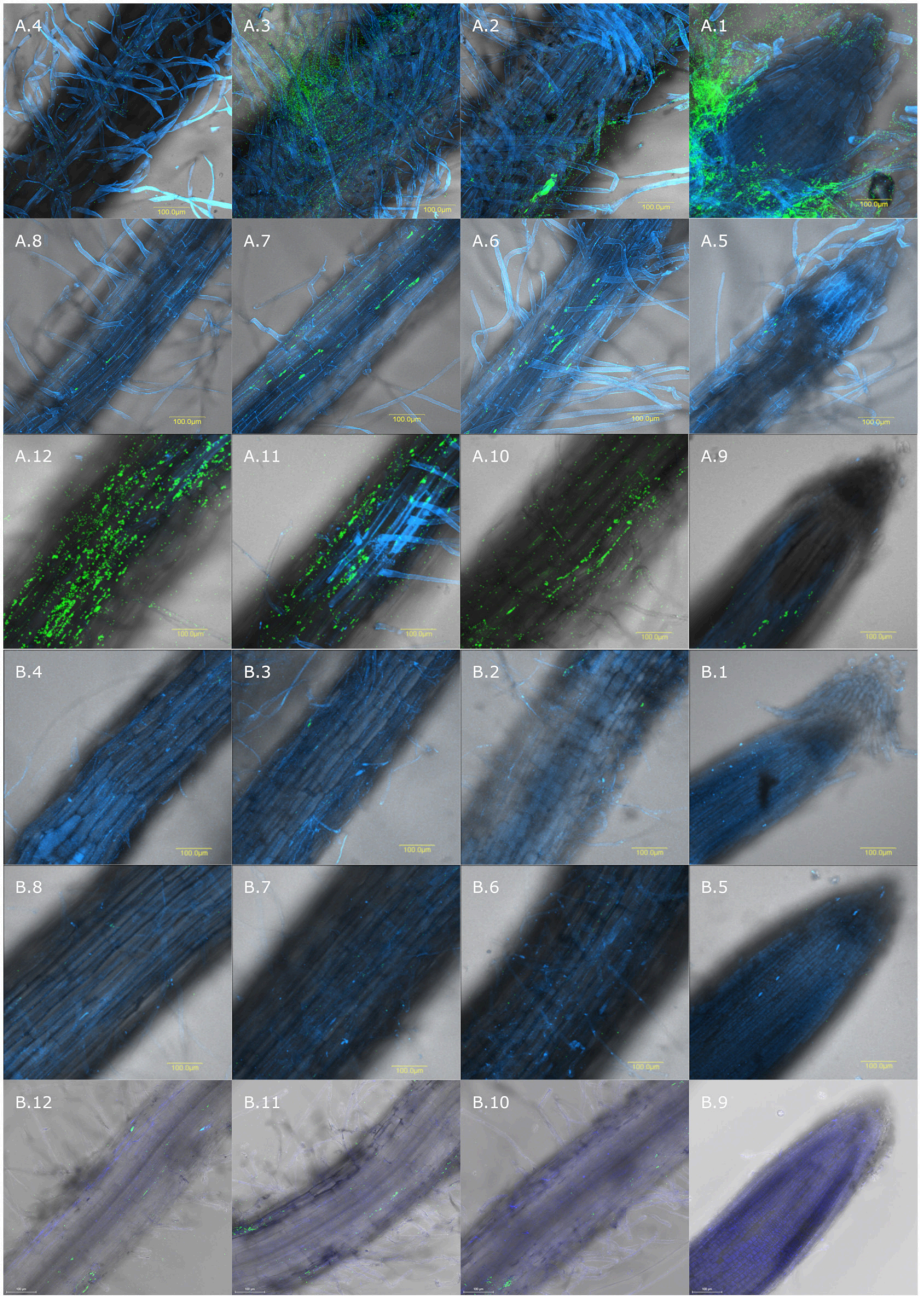
Spatial distribution of colonization on wheat and cucumber roots evaluated by CLSM. Wheat and cucumber roots were sampled after 10–12 days of growth in soil inoculated with GFP labeled isolates. Root is labeled in blue using Hoechst dye and GFP labeled bacteria are shown in green. Wheat roots inoculated with: **(A.1–A.4)** isolate NT0124; **(A.5–A.8)** NT0128; **(A.9–A.12)** NT0133; Cucumber roots inoculated with: **(B.1–B.4)** NT0124, **(B.5–B.8)** NT0128; **(B.9–B.12)** NT0133. (**1**) In all the panels; (1) root tip, (**2**) 0.5 cm from the root tip (**3**) 1 cm from root tip and (**4**) 1.5 cm from the root tip. The different sections of each isolate in both plants, were taken from the same root, starting from the tip toward the upper 1.5 cm. The pictures represent multiple roots from at least two different individual experiments.

In order to successfully utilize bacteria in the root, it is important to understand why one species is found in one plant and not in another. We hypothesized that the wheat-derived isolates will better colonize wheat roots compared to cucumber. In addition, we hypothesized that an isolate with higher biofilm formation ability will also be a better root colonizer. We examined and followed the ability of the three isolates to colonize wheat and cucumber roots by two methods: quantitatively, using qPCR analysis (Figure 3) and qualitatively on root topography, using CLSM (Figure 4).

Soil inoculated with the three GFP labeled strains, as described in the methods section, was used to grow either wheat or cucumber seedlings. The roots of 10–12 days old seedlings were used for assessing bacterial colonization. Root colonization was quantified using qPCR targeting unique GFP gene sequences in each isolate and primers that target a plant gene *(tef*) representing plant cells number. qPCR results (Figure 3) showed that the abundance of all three isolates, normalized to *tef* copy number, was significantly affected by the plant type (Two way ANOVA *p* < 0.005, F ratio = 8.6). All three isolates colonized wheat, on average at a level of 34 bacterial cells per root cell, while isolates NT0124 and NT0128 showed a slightly higher GFP copy number per root cell. Normalized GFP copy number on cucumber root was at least 10 times lower than wheat root, suggesting preferential colonization of isolates. Isolates NT0124 and NT0128 seem to be the best wheat root colonizers.

Colonization of the GFP labeled strains was analyzed by using CLSM (Figure 4). CLSM sections of each isolate in both plants were taken from the same root starting from the tip toward the upper 1.5 cm. CLSM images further demonstrated wheat over cucumber colonization preference, supporting the qPCR results. Additionally, CLSM showed that each of the isolates had a different spatial distribution on wheat root. Isolate NT0128 did not show any preference toward a specific niche of wheat roots (Figures 4A.5–A.8), exhibiting a root wide colonization distribution. In contrast, NT0124 and NT0133, which were better wheat root colonizers, showed preference in specific zones. NT0124 colonized preferentially the zones closest to the roots tip (Figures 4A.1–A.4). Interestingly, this isolate also showed high preference toward root hairs and formed massive biofilms (Figure S2). Isolate NT0133, on the other hand, showed colonization preference toward the zone distant from the root tip (1–1.5 cm from the root tip) (Figures 4A.9–A.12). However, all three isolates were barely found along cucumber roots at the studied zones (Figures 4A.9–A.12,B.1–B.4).

## Discussion

Theories in ecology suggest that forces such as availability of resources and competition will restrict the number of species sharing the same niche (Hardin, 1960; Leibold and McPeek, 2006; Ghoul and Mitri, 2016). The root environment has high diversity of bacteria; many of them compete for space and nutrients obtained from the soil, but more so from the root itself. In addition, root deposits are known to differ by plant species and along the root (Badri and Vivanco, 2009; Berendsen et al., 2012; Bulgarelli et al., 2012). Several studies suggest that plant host can influence the composition and activity of its root microbiome. For example, cereals roots were shown to often be rich in members of the genus *Pseudomonas* (Ofek et al., 2014; Rascovan et al., 2016). However, little is known on the spatial distribution and preference of root species coexisting in the same niche.

Three dominant *Pseudomonas* strains were isolated from wheat roots, to examine their pattern of colonization and specifically their niche preference. Two isolates (NT0124, NT0128) were closely related to the *P. stutzeri* group, members of which were previously shown to colonize plant roots and have great importance in the nitrogen requirement of plants and in degradation of aromatic compounds (Dekkers et al., 1998; Lugtenberg and Dekkers, 1999; Rediers et al., 2009; Silby et al., 2011). The third isolate NT0133, was related to *P. fluorescens*, which often serves as a model organism for the description of root colonization processes (Rivilla et al., 2013; Ramos et al., 2015). Members of *P. fluorescens* group are often found on plant roots and in soils and some strains of this group are known for their abilities to protect plants from pathogens (Silby et al., 2011) and promote wheat growth (Capper and Higgins, 1993; Weller, 2007). All three isolates preferentially colonized wheat roots over those of cucumber, suggesting host specificity. Furthermore, similar experiments showed preference to other cereals (barley and maize) in comparison to soybeans (data not shown). Our results show that the three isolates differ in motility and biofilm formation traits, both considered important in root colonization. Surprisingly, those traits appear not to predict which isolate will succeed in colonizing plants roots. It has been shown here, and in other studies (Lugtenberg and Dekkers, 1999; Lugtenberg et al., 2001; Velázquez-Sepúlveda et al., 2012; Ofek-Lalzar et al., 2014; Rascovan et al., 2016), that the plant host appears to determine which bacterial species may succeed in its colonization. In the current study, we suggest that the plant host may also have a role in shaping the particular location of colonization. Our findings strongly suggest that the exact localization of colonization on the roots can vary, even between related bacterial species. One would assume that three related organisms will compete for nutrients and space and thus not inhabit the same niche (Hardin, 1960; Hibbing et al., 2010; Ghoul and Mitri, 2016). Indeed, these different characteristics in motility and biofilm formation and colonization traits may have a role in shaping the spatial distribution and coexistence of these related isolates on the roots. The preferred localization of a bacterial species along the root may indicate differences in available resources. It is assumed that different root deposits may be abundant at the various root zones, e.g., near the root tip, near the zone of maturation, which has numerous root hairs, or in microcolonies co-colonizing with other microorganisms (Walker et al., 2003; Badri and Vivanco, 2009; Dennis et al., 2010; Sasse et al., 2017). In addition to the metabolic capabilities, other bacterial traits might reason the distinct spatial distribution of the strains. The highly motile isolate NT0124 was found to preferentially colonize the zone near the tip. Succeeding at this location may be explained by this strains ability to “chase” the tip as the root elongates. As plant root grows, the root tip is pushed forward, and thus motile bacteria will have an advantage in consistently colonizing the root tip, a major zone of exudate secretion (Badri and Vivanco, 2009). NT0128 showed similar abundance to NT0124 on wheat roots, however, without preference on zone of colonization. The difference between the two isolates could result from variety of traits. For example, the possibility that NT0128 is less selective in resource utilization, which may expand its colonization possibilities. Such hypothesis is supported by difference in their genomes; the two isolates share only 707 ORFs out of total of 4071 of NT0124 and 4202 of NT0128 (Figure S1) and although both isolates were identified as *P. stutzeri* they belong to two different phylogenetic clades (Figure 1). In addition, on the plate assay NT0124 motility was slightly faster than NT0128. In the root environment this phenomenon could be more pronounced, giving NT0124 an advantage in continuously moving niche such as the root tip. Lastly, isolate NT0133 showed high competence and preference in colonizing the zone distant from the wheat root tip, suggesting adaptation to this niche. Ultimately, the data presented here demonstrates that understanding bacterial motility may be important in predicting the niche preference of a specific strain.

The importance of flagella-dependent motility to root colonization is still under debate. Some studies reported that non-motile mutants of *Pseudomonas* are not impaired in root colonization of wheat and soybean (Howie et al., 1987; Scher et al., 1988; Lugtenberg et al., 2001), while others showed that some non-motile mutants were severely impaired in colonization of potato and tomato root (De Weger et al., 1987; Lugtenberg et al., 2001). This discrepancy in results might be explained now by authors disregard to micro-niche preferences of the *Pseudomonas* strains they used. Motility is also required for the early stages of biofilm formation and for surface attachment. In the biofilm state the bacteria are better protected and this trait was shown to be especially important for adaptation to living on roots (Walker et al., 2004). Our strains successfully form biofilm; NT0124 and NT0128 biofilm formation on polystyrene surface was at levels equivalent to that of the commonly used biofilm model PAO1 and indeed formed massive biofilm on wheat roots. While isolate NT0133 form less biofilm on abiotic polystyrene surface, this did not affect its ability to adhere and colonize the non-moving parts of wheat roots. This suggests that successfull root colonization is not influenced solely by motility and biofilm formation competence.

Our results demonstrate that different areas along roots can favor different species. Even related species can have significant variation in their spatial colonization patterns, governed by both niche and bacterial characteristics. These findings can help our understanding of plant-bacteria interaction and pave the road for studies that will elucidate the mechanisms by which bacteria and plants select each other. This understanding may be of importance in future attempts to interfere with root microbiomes, construct and design effective synthetic communities.

## Author Contributions

NT, YH, and DM designed the experiments. NT performed the experiments. SF analyzed and the genomes sequences. All authors jointly wrote the manuscript.

### Conflict of Interest Statement

The authors declare that the research was conducted in the absence of any commercial or financial relationships that could be construed as a potential conflict of interest.

## Supplementary Material

Figure S1 Venn diagram depicting numbers of unique and shared orthologs predicted open reading frames (ORFs). Orthology was determined using ProteinOrtho software. The number of ORFs corresponding to the number of orthologs is in parenthesis.

Figure S2 Enlargement of zone showed in picture A.1 (0.5 cm from wheat root tip). Wheat roots were sampled after 10–12 days of growth in soil and soil was inoculated with isolate NT0124 which formed massive biofilm on wheat hairs.
