# Supplementary figures and images for: Host Specificity and Spatial Distribution Preference of Three Pseudomonas Isolates
Source: Front Microbiol. 2019 Jan 10;9:3263. doi: 10.3389/fmicb.2018.03263 (PMC6335278; doi:10.3389/fmicb.2018.03263)

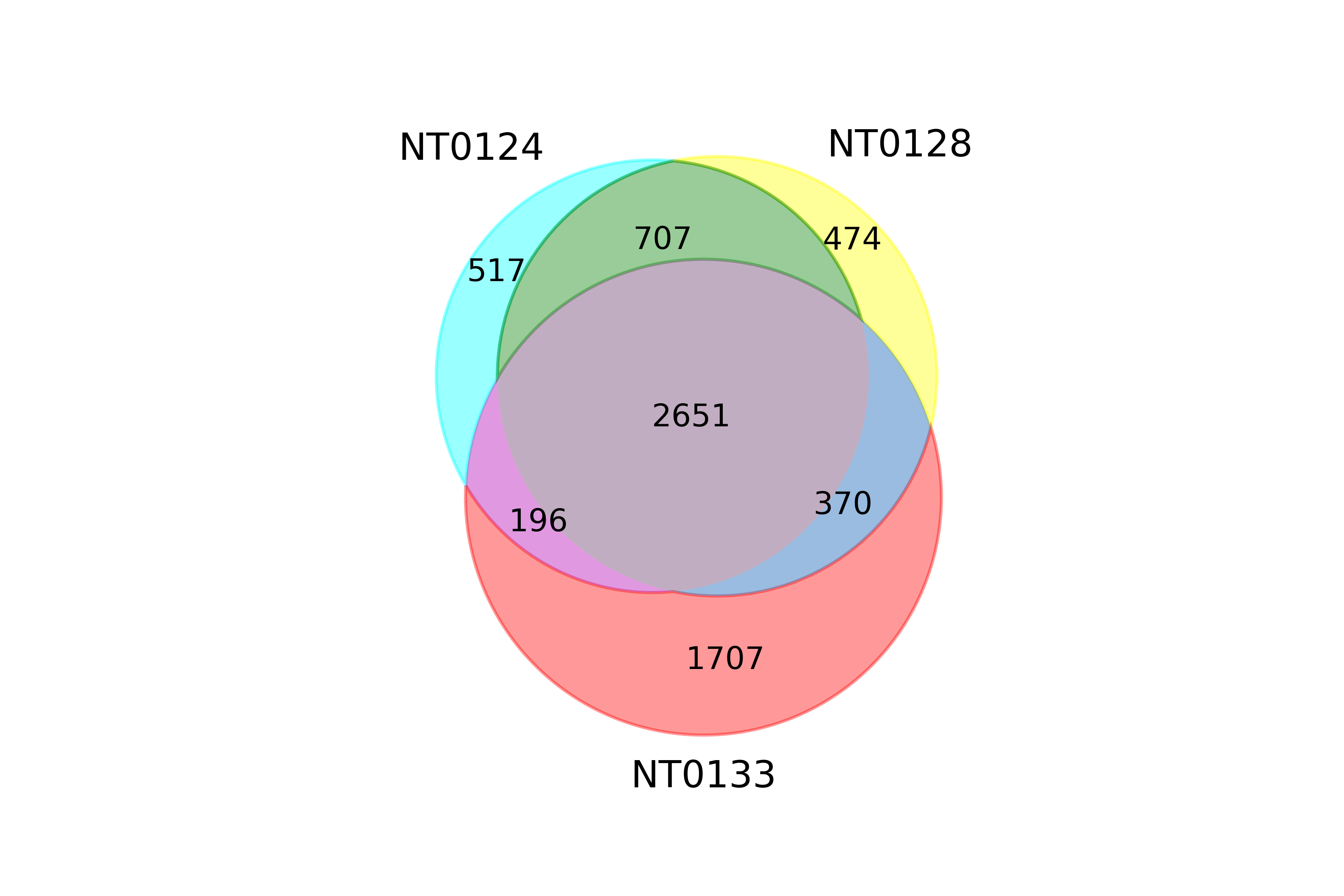

Supplement: Figure S1 — Venn diagram depicting numbers of unique and shared orthologs predicted open reading frames (ORFs). Orthology was determined using ProteinOrtho software. The number of ORFs corresponding to the number of orthologs is in parenthesis. [file Data_Sheet_1.zip › Figure_S.1.tif]

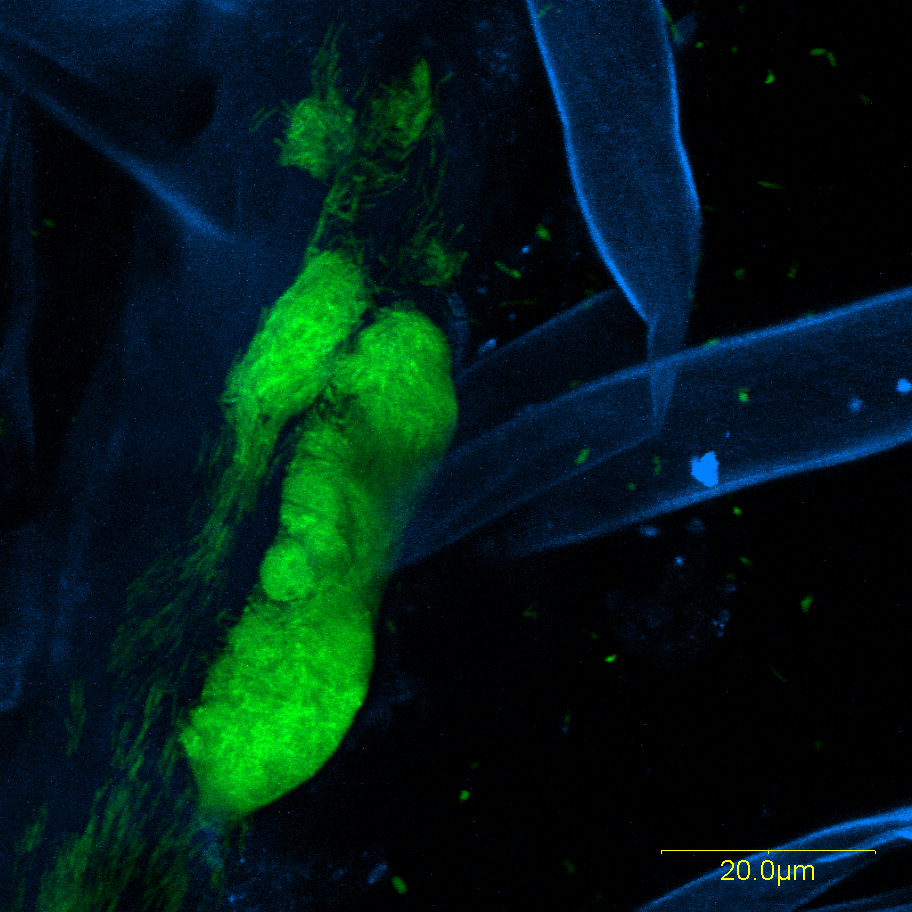

Supplement: Figure S1 — Venn diagram depicting numbers of unique and shared orthologs predicted open reading frames (ORFs). Orthology was determined using ProteinOrtho software. The number of ORFs corresponding to the number of orthologs is in parenthesis. [file Data_Sheet_1.zip › figure_S.2.tif]
